# Supplementary material for: A Shift in Asthma Treatment According to New Guidelines: An Evaluation of Asthma Patients’ Attitudes towards Treatment Change
Source: Int J Environ Res Public Health. 2023 Feb 16;20(4):3453. doi: 10.3390/ijerph20043453 (PMC9958739; doi:10.3390/ijerph20043453)
Supplement: Supplementary file 1 [file ijerph-20-03453-s001.zip › ijerph-2116053-supplementary.pdf]

JBI CRITICAL APPRAISAL CHECKLIST FOR QUALITATIVE RESEARCH

Reviewer_______________________________________ Date________________________________

Author__Sara Sommer Holst________________________Year_2023_____ Record Number_ijerph-2116053_

	Yes	No	Unclear	Not applicable
1. Is there congruity between the stated philosophical perspective and the research methodology?	☒	☐	☐	☐
2. Is there congruity between the research methodology and the research question or objectives?	☒	☐	☐	☐
3. Is there congruity between the research methodology and the methods used to collect data?	☒	☐	☐	☐
4. Is there congruity between the research methodology and the representation and analysis of data?	☒	☐	☐	☐
5. Is there congruity between the research methodology and the interpretation of results?	☒	☐	☐	☐
6. Is there a statement locating the researcher culturally or theoretically?	☒	☐	☐	☐
7. Is the influence of the researcher on the research, and vice- versa, addressed?	☒	☐	☐	☐
8. Are participants, and their voices, adequately represented?	☒	☐	☐	☐
9. Is the research ethical according to current criteria or, for recent studies, and is there evidence of ethical approval by an appropriate body?	☒	☐	☐	☐
10. Do the conclusions drawn in the research report flow from the analysis, or interpretation, of the data?	☒	☐	☐	☐

Overall appraisal: Include ☒ Exclude ☐ Seek further info ☐

Comments (Including reason for exclusion)

____

____
